# Self-reported work ability of Norwegian women in relation to physical and mental health, and to the work environment

**DOI:** 10.1186/1745-6673-3-8

**Published:** 2008-04-22

**Authors:** Migle Gamperiene, Jan F Nygård, Inger Sandanger, Bjørn Lau, Dag Bruusgaard

**Affiliations:** 1 Department of General Practice and Community Medicine, University of Oslo, Norway; 2 Helse Øst Health Services Research Unit, Akershus University Hospital, Faculty of Medicine, University of Oslo, Norway; 3 Helse Øst Health Services Research Unit, Akershus University Hospital, Faculty of Medicine, University of Oslo, Norway; 4 National Institute of Occupational Health, Oslo, Norway

## Abstract

**Objectives:**

To examine the self-reported level of work ability among female employees and the relationship between work ability and demographic characteristics, physical health, mental health, and various psychosocial and organizational work environment factors.

**Methods:**

Participants were 597 female employees with an average age of 43 years from urban and rural areas in Norway. Trained personnel performed a structured interview to measure demographic variables, physical health, and characteristics of the working environment. Mental health was assessed using the 25-item version of the Hopkins Symptoms Checklist (HSCL-25). Work ability was assessed using a question from the Graded Reduced Work Ability Scale.

**Results:**

Of the 597 female employees, 8.9% reported an extremely or very reduced ability to work. Twenty-four percent reported poor physical health and 21.9% reported mental distress (≥ 1.55 HSCL-25 cut-off). Women, who reported moderately and severely reduced work ability, did not differ a lot. Moderately reduced work ability increased with age and was associated with physical and mental health. Severely reduced work ability was strongly associated only with physical health and with unskilled occupation. Of eight work environment variables, only three yielded significant associations with work ability, and these associations disappeared after adjustment in the multivariate analysis.

**Conclusion:**

Results indicate that ageing, in addition to poor self-reported physical health and unskilled work, were the strongest factors associated with reduced work ability among female employees. Impact of work environment in general was visible only in univariate analysis.

## Introduction

Work ability is a multi-faceted and multi-determined concept not only associated with health, but also with competence, values, the working environment, and social relations [1]. Level of work ability in the working population can predict both future permanent disability [2] and duration of sick leave absences [3].

A high rate of work disability in the workforce in western countries gives reason for concern [4,5] and underscores the importance of identifying and modifying potential risk factors. Various demographic, physical, psychosocial, and organizational factors have been investigated, yet results are often inconclusive. Studies have shown that individuals over the age of 45 deteriorate about 1.5% per year in work capacity [6,7] and work disability increases with increasing age [5,8,9].

The connection between work ability and physical and mental health has been established in several studies [10-12]. Results have found self-perceived poor health to be the strongest risk factor for poor working ability, and this association remains significant even after controlling for age. Other indicators of health status, such as overweight, have also demonstrated a negative association with working ability [9].

Although physical health has well-substantiated ramifications for future disability, studies also emphasize the importance of considering other factors in addition to health to understand and possibly prevent disability. Psychosocial factors, such as mental demands at work, controllability of work, time pressure, and leadership/management are factors affecting work ability [2,4,13]. Emotional stress resulted in reduced work ability among teachers [14] and the perception of job insecurity was associated with poor work ability in a study of the public sector employees in Sweden [15]. In contrast, positive experiences such as effectively utilizing one's abilities and skills, having the potential for professional development, exerting influence/control at work, and job satisfaction have been associated with good work ability [9,10,16].

The effects of particular work characteristics on work ability appear to vary across occupation, rendering type of occupation an important factor to consider. For example, reduced work ability is more prevalent among blue-collar workers in both genders [17]. Torgen and Kilbom [18] found that in contrast to men, who experienced an increase in skilled work and decreased physical loads, the proportion of female unskilled workers has increased in the Swedish population over a 24-year period, and their physical loads have either remained unchanged or increased.

The influence of psychosocial and work environmental factors on work ability, especially among female low-status occupations, is not fully understood. Therefore, we examined if: 1) work ability decreases with age and poor self-reported physical and mental health, and 2) work ability decreases with increasing physical, psychosocial and organizational problems within the working environment, with differential effects according to type of occupation (skilled versus non-skilled).

## Subjects and methods

### Sample, data selection

Participants were originally recruited for a population-based study initiated in 1990 to examine issues related to mental health within two geographically diverse areas in Norway. Further details regarding the study are presented elsewhere [19]. A total of 2727 adults from the two study regions (Oslo, urban and Lofoten, rural) were randomly selected by Statistics Norway. Of these, 2014 (74%) individuals participated, who were representative of age and gender for the general Norwegian population. Ten years later, 65% (N = 1300) were available for a follow-up study, plus 1000 new randomly selected participants were added to increase the number of younger individuals and immigrants within the sample. Of the 2300 invited, 1691 persons (803 men and 888 women) participated in the follow-up. For the present study, all women who reported having paid employment were selected for the analyses (597 of 888). All data were collected using structured face-to-face interviews by trained interviewers.

### Dependent variable: work ability (WA)

Work ability was assessed by the question "How do you estimate your work ability today?" This question was selected from the Graded Reduced Work Ability Scale, which was constructed for the Norwegian Ministry of Health and Social Affairs [20]. Responses are scored on a scale from 1 (extremely reduced) to 6 (not reduced at all). Work ability was recoded into three categories: severe reduced (1–2) work ability, moderately reduced (3–5) and not reduced (6) work ability.

### Independent variables

Individual factors were age, ethnicity, marital status, and residence. Ethnicity was dichotomized according to whether the woman was born in Norway or not. Marital status was dichotomized according to whether the woman was married/cohabitating or not. Place of residence variable was categorized according to urban (Oslo), rural (Lofoten), or other (moved elsewhere). Working time was measured by working days per week. Four days and more per week were coded as full time work, and one day and less – part time work.

Physical health was assessed by the question "How satisfied are you with your physical health and well-being?" The score ranged from 1 (extremely satisfied) to 7 (extremely dissatisfied). The item was recoded into three categories: satisfied (from 1 to 3), partially satisfied (4), and dissatisfied (from 5 to 7). Weight and height were registered and body mass index (BMI) was calculated using the formula weight (kg) divided by height (m) squared. Consistent with established criteria, obesity was defined as a BMI of ≥ 30 kg/m^2 ^and participants were classified as obese or non-obese.

Mental health status was assessed by Hopkins Symptoms Checklist (HSCL-25) [21]. The HSCL-25 is a 25-item self-report questionnaire about the presence and intensity of anxiety and depression symptoms during the previous week. Items are scored on a scale from 1 (no distress) to 4 (extremely distressed). The HSCL-25 score was calculated as the sum score of items divided by number of items answered. To be counted as a valid HSCL-25 score, at least 13 items had to be answered. A score from 1 to 1.54 was defined as "no mental distress," from 1.55 to 1.74 as a "mild mental distress," and score equal to or larger than 1.75 was defined as "severe mental distress" [19,22].

The work environment was assessed based on seven questions originally used in work/life household surveys in Norway [23]. Physical and mental strain at work were assessed by the questions, "Is your job so physically strenuous that you are often physically tired after work?" and "Does your work demand so much concentration and attention that you often feel exhausted after work?" Job stress was assessed by the question, "Is there a lot of stress at your workplace?" Scores ranged from 1 (never) to 4 (almost always) and were recoded into two categories: never (1) and sometimes/all the time (from 2 to 4). Level of decision-making at work was assessed by the question, "To what extent do you personally make decisions about your work?" Scoring ranged from 1 (I decide myself) to 4 (immediate superior decides what and how), and the score was then recoded into two categories: decide myself (1) and partial control over decisions/immediate supervisor decides what and how (from 2 to 4). Opportunity for effectively utilizing skills at work was assessed by the question, "Do you feel that you utilize your skills and abilities at work?" Scores ranged from 1 (yes) to 4 (no). The answers were recoded to: yes (1) and partially/no (from 2 to 4). Job insecurity was assessed by the question, "Is there any risk that you might lose your current job in the near future?" The score ranged from 1 (yes, absolutely) to 5 (no, very unlikely) and responses were dichotomized into yes (from 1 to 3) and no (from 4 to 5). Job satisfaction was assessed by the question "How satisfied are you with your job?" The score ranged from 1 (very satisfied) to 7 (not satisfied at all) and was recoded to: satisfied (from 1 to 3), partially satisfied/dissatisfied (4–7). These seven work environment variables were included both separately and as a sum score. The work environment sum score (0 to 13) was recoded into three categories: good (0 – 3), average (4 – 6), and poor (7 – 13).

Occupation was registered as free-text, and then recoded according to the Standard Classification of Occupations and dichotomized into either 'skilled' or 'unskilled' work [24].

### Statistical methods

A chi-square test was used to analyse differences in proportions and Pearson's correlation was calculated for all the variables. Poisson univariate regression was calculated with 95% confidence intervals (CI) to estimate risk ratios (RR) between the dependent variable, reduced work ability, and the selected independent variables (demographic, physical health and well-being, mental health, and work environment). Finally, significant risk factors were entered into a Poisson regression model for both categories of dependent variable. The significance level was set at p < .05. All analyses were performed using STATA, version 8.2.

## Results

The average age of the sample was 43.2 years and the majority were married or cohabitating (70.2%). Approximately 42% resided in Oslo (urban) and 43.7% in Lofoten (rural). The vast majority (91.2%) was born in Norway. Fifty-seven percent were employed in skilled jobs and 43% had unskilled work. The majority (73%) worked full time. Twenty-four percent reported partial satisfaction or dissatisfaction with their physical health and well-being and 21.9% had HSCL-25 scores equal to or above cut-off of 1.55. Continuous physically or mentally strenuous work was reported by 8.5% and 9.9% of the sample, respectively. Job insecurity was reported by 13.6% of the women and 15.1% were only partially satisfied or were dissatisfied with their job.

### Work ability and potential risk factors

Table 1 illustrates the distribution of self-reported work ability according to age (years). Of the 597 female employees, 8.9% reported an extremely or very reduced ability to work.

**Table 1:** Distribution of self-reported work ability and age (years) among female employees in Norway (N = 597)

Work Ability (WA)								
Age (years)	Extremely reduced	Very reduced	Moderately reduced	Not particularly reduced	Slightly reduced	Not reduced at all	Total	
	n (%)	n (%)	n(%)	n (%)	n (%)	n (%)	Missing (n)	N

≤ 29	2 (4.6)	1 (2.3)	3 (6.8)	6 (13.6)	7 (15.9)	25 (56.8)	-	44
30–39	2 (1.2)	7 (4.1)	20 (11.8)	14 (8.3)	41 (24.3)	81 (47.9)	4	169
40–49	5 (2.1)	15 (6.4)	25 (10.6)	24 (10.2)	77 (32.6)	87 (36.9)	3	236
50–67	6 (4.1)	15 (10.2)	31 (21.1)	20 (13.6)	38 (26.6)	33 (22.5)	4	113
Missing (n)	-	-	-	-	-	-	1	34
Total	15 (2.5)	38 (6.4)	79 (13.2)	64 (10.7)	163 (27.3)	226 (37.9)	12	597

Table 2 shows the level of self-reported work ability categorized into not reduced, moderately reduced and severely reduced according to demographic variables, health status, and psychosocial and organizational aspects of the work environment. The Pearson's chi-square to test proportional differences and the univariate rate ratios (RR; 95% CI) for level of work ability are also presented. Increasing age demonstrated a significant effect on the proportion of women reporting a reduction in work ability. The proportion of women reporting no reduction in work ability fell from 51.0% among the youngest to 19.4% among those 50 years and older.

**Table 2:** Distribution and Poisson univariate associations between work ability and demographic variables, physical and mental health, and work environment characteristics among female employees in Norway (N= 597)

	Work Ability (WA)							
	Not reduced WA n = 222	Moderately reduced WA n = 301	Severely reduced WA N = 51		Moderately reduced WA		Severely reduced WA	
	%	%	%	N	RR	CI 95%	RR	95% CI

Demographic variables				585				
**Age **(years) (p < 0.001)								
18 ≤ 29	51.0	43.6	5.4		1 (ref.)		1 (ref.)	
30–39	37.1	54.6	8.3		1.3	0.9 – 1.7	1.9	0.9 – 4.0
40–49	24.6	59.1	16.4		**1.5**	1.1 – 2.1	**4.2**	1.9 – 8.9
50–67	19.4	71.0	9.7		**1.7**	1.1 – 2.7	3.5	0.9 – 12.5
Missing	-	-	-	-	-			
								
**Ethnicity **(p > 0.05)				585				
Norwegian	39.0	52.4	8.6		1 (ref.)		1 (ref.)	
Not ethnic Norwegian	33.3	52.8	13.9		1.1	0.7 – 1.7	1.6	0.7 – 4.1
Missing	-	-	-					
								
**Marital status **(p > 0.05)				564				
Married/cohabiting	40.3	52.0	7.6		1 (ref.)		1 (ref.)	
Not married/not cohabiting	35.2	53.8	11.0		1.1	0.8 – 1.4	1.5	0.8 – 2.7
Missing	20.0	50.0	30.0					
								
**Residence **(p > 0.05)				585				
Oslo (urban)	38.9	52.7	7.8		1 (ref.)		1 (ref.)	
Lofoten (rural)	37.9	53.1	9.0		0.9	0.7 – 1.4	1.5	0.7 – 3.3
Other	38.4	49.3	12.3		1.0	0.8 – 1.3	1.2	0.6 – 2.1
Missing	-	-	-					
								
**Occupation **(p > 0.05)				571				
Skilled	41.2	52.4	6.5		1 (ref.)		1 (ref.)	
Not skilled	35.2	52.6	12.7		1.1	0.8 – 1.3	**1.9**	1.1 – 3.3
Missing	25.0	50.0	25.0					
								
**Working time **(p > 0.05)				569				
Part time	34.8	56.5	8.7		1 (ref.)		1 (ref.)	
Full time	40.4	51.3	8.3		0.9	0.7 – 1.2	0.9	0.4 – 1.6
Missing	26.7	46.7	26.7					
								
Physical and mental health								
**How satisfied are you with your physical health and well-being? **(p < 0.001)				581				
Satisfied	47.4	49.3	3.3		1 (ref.)		1 (ref.)	
Partially satisfied	15.3	72.5	12.2		**1.6**	1.2 – 2.1	**6.9**	3.2 – 14.7
Dissatisfied	4.8	38.1	57.1		**1.7**	1.0 – 2.9	**14.4**	7.4 – 27.8
Missing	25.0	50.0	25.0					
								
**Obesity **(p < 0.001)				552				
BMI < 30	40.0	52.6	7.4		1 (ref.)		1 (ref.)	
BMI ≥ 30	28.0	58.0	14.0		1.2	0.8 – 1.7	2.1	0.9 – 4.8
Missing	31.8	36.4	31.8					
								
**Mental health **(p < 0.001)				585				
No mental distress	46.2	47.5	6.3		1 (ref.)		1 (ref.)	
Light mental distress	24.1	64.8	11.1		**1.4**	1.0 – 2.1	**2.7**	1.1 – 6.4
Severe mental distress	2.8	73.6	23.6		**1.9**	1.4 – 2.6	**7.5**	4.1 – 13.7
Missing	-	-	-	-	-			
								
Work environment								
**Physically strenuous work **(p < 0.05)				562				
Never	44.6	47.9	7.5		1 (ref.)		1 (ref.)	
Sometimes/All the time	33.9	56.6	9.5		1.2	0.9 – 1.5	1.5	0.9 – 2.7
Missing	25.0	50.0	25.0					
								
**Mentally strenuous work **(p < 0.05)				562				
Never	50.6	41.8	7.9		1 (ref.)		1 (ref.)	
Sometimes/All the time	36.8	55.6	8.7		1.3	0.9 – 1.8	1.4	0.6 – 3.2
Missing	25.0	50.0	25.0					
								
**Workplace stress **(p > 0.05)				562				
Never	32.8	56.9	10.3		1 (ref.)		1 (ref.)	
Sometimes/All the time	39.7	52.0	8.3		0.9	0.6 – 1.3	0.7	0.3 – 1.7
Missing	25.0	50.0	25.0					
								
**Do you feel that you utilize your abilities at work? **(p < 0.05)				558				
Yes	44.2	49.8	6.0		1 (ref.)		1 (ref.)	
Partially/No	33.2	55.2	11.6		1.2	0.9 – 1.4	**2.2**	1.2 – 3.9
Missing	25.0	56.3	18.8					
								
**To what extent do you personally make decisions about your work? **(p > 0.05)				562				
I decide myself	40.9	53.8	5.3		1 (ref.)		1 (ref.)	
I partly decide/Immediate supervisor decides what and how	38.4	52.1	9.5		1.0	0.8 – 1.3	1.7	0.8 – 3.9
Missing	25.0	50.0	25.0					
								
**Are you satisfied with your work? **(p < 0.000)				582				
Satisfied	43.3	50.5	6.2		1 (ref.)		1 (ref.)	
Partly satisfied/Not satisfied	12.6	63.2	24.1		**1.5**	1.2 – 2.1	**5.3**	3.0 – 9.2
Missing	50.0	50.0	-					
								
**Is there any risk that you might lose your job? **(p > 0.05)				562				
No	38.9	52.4	8.7		1 (ref.)		1 (ref.)	
Yes	39.5	53.1	7.4		0.9	0.7 – 1.4	0.8	0.4 – 2.0
Missing	25.0	50.0	25.0					
								
**Work environment (total) **(p < 0.01)				556				
Good	52.2	43.4	4.4		1 (ref.)		1 (ref.)	
Average	35.5	55.6	8.9		**1.3**	1.0 – 1.8	**2.6**	1.2 – 5.7
Bad	22.2	60.5	17.3		**1.6**	1.1 – 2.3	**5.6**	2.4 – 13.4
Missing	27.8	55.6	16.7					

Note: Data on Work Ability (WA) were missing for 12 cases (2,01%)

Work in unskilled occupations (RR = 1.9) and feeling that women do not utilize her abilities at work (RR = 2.2) was associated with severe reduced work ability.

Both partially satisfaction and dissatisfaction with physical health and well-being were associated with moderately reduced work ability (RR = 1.6 and RR = 1.7) and severely reduced work ability (RR = 6.9 and RR = 14.4).

Mild and severe mental distress was also associated with both moderately (RR = 1.4 and RR = 1.9) and severely (RR = 2.7 and RR = 7.5) reduced work ability in the univariate analyses.

Reporting job dissatisfaction was associated with a moderately and severely reduction in work ability compared to satisfied employees (RR = 1.5 and RR = 5.3). Analysis with sum score for work environment showed that women who reported an "average" or "poor" working environment had a higher risk of reporting both moderately reduced work ability (RR = 1.3 and RR = 1.6) and severely reduced work ability (RR = 2.6 and RR = 5.6) than employees who were satisfied with the overall work environment.

Neither ethnicity, marital status, residence, working time, BMI, nor physically and mentally strenuous work, workplace stress, level of decision-making, and job insecurity were associated with work ability.

When examining the pattern of correlations among variables, several significant (p < 0.01) correlations existed: job satisfaction and mental health (r = 0.39), job satisfaction and physical health/well-being (r = 0.36), mental and physical health/well-being (r = 0.37), and workplace stress and mental strain (r = 0.37; data not shown in tables). There was a significant difference in the proportion of women reporting "job dissatisfaction" among those who were satisfied with their physical health/well-being and those who were not (2.3% versus 25.9%, respectively; p < 0.001). Similar results were found for mental distress (1.8% versus 22.4%, respectively; p < 0.001).

In the multivariate regression model, we included the variables, which had significant elevated incidence ratios in univariate analyses (see Table 3). In the multivariate analysis, the following variables were significant for moderately reduced work ability: age groups 30–39, 40–49 years and 50+ years (RR = 1.3, 1.5 and 1.8) and severe mental distress (RR = 1.5). Unskilled occupation (RR = 1.9) and partially satisfaction and dissatisfaction with physical health remained significant for severe reduced work ability (RR = 5.1 and IR = 9.5).

**Table 3:** Poisson regression analysis of work ability according to demographic variables, physical and mental health, and of the work environment among female employees in Norway (N = 597)

Risk factors	Moderately reduced Work Ability (WA)		Severely reduced Work Ability (WA)	
	RR	95% CI	RR	95% CI
				
Age (years)				
≤ 29	1 (ref.)		1 (ref.)	
30–39	**1.3**	1.0 – 1.8	1.6	0.7 – 3.5
40–49	**1.5**	1.1 – 2.1	1.5	0.6 – 3.7
50 – 67	**1.8**	1.1 – 2.9	2.3	0.4 – 12.5
				
Occupation				
Skilled	1 (ref.)		1 (ref.)	
Not skilled	0.9	0.8 – 1.3	**1.9**	1.1 – 3.5
				
How much are you satisfied with your physical health and well-being?				
Satisfied	1 (ref.)		1 (ref.)	
Partially satisfied	**1.4**	1.0 – 1.9	**5.1**	2.2 – 11.9
Dissatisfied	1.6	0.9 – 2.7	**9.5**	3.9 – 23.2
				
Mental health				
No mental distress	1 (ref.)		1 (ref.)	
Light mental distress	1.3	0.9 – 1.9	1.7	0.6 – 4.8
Severe mental distress	**1.5**	1.1 – 2.1	1.3	0.6 – 3.1
				
Do you feel that you utilize your abilities at work?				
Yes	1 (ref.)		1 (ref.)	
Partially/No	1.0	0.8 – 1.3	1.0	0.5 – 2.3
				
Are you satisfied with your work?				
Satisfied	1 (ref.)		1 (ref.)	
Partly satisfied/Not satisfied	1.0	0.7 – 1.5	1.4	0.5 – 3.6
				
Work environment				
Good	1 (ref.)		1 (ref.)	
Average	1.3	0.9 – 1.8	1.6	0.7 – 4.0
Bad	1.4	0.9 – 2.1	1.4	0.4 – 4.9

## Discussion

We found that moderately reduced work ability increased with age and was associated with physical and mental health. Severely reduced work ability was associated with unskilled occupation and strongly with physical health. Of the eight work environment variables, only three yielded significant associations with reduced work ability, although these associations disappeared after adjustment in the multivariate analysis. Women, who reported moderately and severely reduced work ability did not differed a lot. They reported associations with the same health and work environment variables: utilization of abilities and satisfaction at work, and work environment in general. However, women in unskilled occupations reported severely reduced work ability.

### Methodological considerations

Our sample was randomly drawn from an urban and a rural area in Norway, and included only women who reported having paid employment at the time of our study. Unhealthy individuals are more likely to exit the workforce with disability pension. Such a selection effect, also called the Healthy Worker Effect, may have weakened the associations between work ability and work environment. The cross-sectional design precludes our ability to draw conclusions regarding the direction of relationships among our study variables. Data were dependent upon the employee's momentary state, which may have biased reporting of health, work ability, and work environment problems. However, all data collection was conducted in the presence of a trained investigator, which may have assisted in overcoming shortcomings.

The question on work ability was chosen based on a study by Haldorsen and colleagues [20,25]. It has been found that self-evaluated work ability correlates significantly with clinically determined musculoskeletal capacity in healthy women [26], which provides some support for the construct validity of our dependent variables [27].

The assessment of physical health also relied upon single-item measurement. However, the feasibility and predictive validity of perceived self-reported health has been demonstrated in several studies [28-30]. The Hopkins Symptom Checklist-25 (HSCL-25) was used for the assessment of mental distress [22]. The HSCL-25 has been validated in this same cohort using the selected thresholds (i.e., HSCL-25 ≥ 1.55 to indicate a possible case and ≥ 1.75 to indicate a probable case), and the instrument is generally considered a good indicator for mental health distress [31-33]. Our selected thresholds are identical to those in other population studies, thus increasing the comparability of our results [22,34].

### Results

Reduced physical and mental health had the strongest impact on work ability among Norwegian female employees. Results appear consistent with findings from Illmarinen et al (1997), who documented that changes in employee health status yielded the strongest impact on work ability [2,4]. Another study found that the level of sick leave during the previous year was a strong predictor of poor work ability [35,36]. In contrast to prior findings, however, obesity was not related to work ability in our study [9]. Off all demographic variables only adverse effects of ageing for moderately and severely reduced work ability were demonstrated. Women over the age 50 years had almost two-times greater association with moderately reduced work ability than women aged 18–29 years. Only age group 40–49 years was associated with a severe reduced work ability. This result might owe to a healthy worker effect, whereby only the healthiest employees survive in unfavourable working conditions. Longitudinal studies have reported similar results regarding ageing and disability. An 11-year follow-up study in Finland found that women over the age of 51 years had the highest annual declining rate in work ability [2]. For women, the physiological and mental changes associated with menopause and a general decline in abilities to cope with stress during older adulthood may partially account for the results [2].

Impaired health might be a result of earlier influence of work environment [2,4,7,10]. Results in our study do not support this as impact of work environment in general on work ability disappears after health outcomes are entered in the regression.

Regarding the work environment, only having the opportunity to utilize one's skills and abilities, and job satisfaction was associated with reduced work ability in the univariate analysis. However, these associations lost significance after controlling for the effects of other variables in the multivariate analysis. The sum score for the work environment variables showed strong association with reduced work ability in the uniariate analysis but lost its significance in the final model. Although weaker than expected, results pertaining to work environment were comparable to prior research by Linberg et al [36]. Additionally, the associations between self-reported ability to utilize one's skills and job satisfaction, and work ability have been established in earlier studies [16,37].

For utilization of one's abilities at work and for work satisfaction, associations with severely reduced work ability demonstrated stronger adverse effects than with moderately reduced work ability in the univariate analysis. These associations were lost in the final adjusted model. The strong correlations between physical health, mental health, and work satisfaction may indicate that physical and mental health mediates the relationship between job satisfaction and work ability. The higher prevalence of physical and mental health problems among the "dissatisfied" group versus the "satisfied" group provides additional support for this assertion. Findings are comparable with prior research by Faragher et al [38].

Somewhat unexpectedly, and in contrast to other studies [4,13,16,36], we did not find a significant relationship between perceived work ability and the opportunity to exert influence and control over decision-making at work, job-security, or workplace stress. Influence and control, in addition to workplace stress, were found to be highly associated with mental distress among women in this sample and may have therefore become insignificant when adjusting for HSCL-25 scores [39].

Owing to the association between unskilled work and physically strenuous work and the established findings on physical demanding work and disability [2], we expected women in unskilled occupations to report more reduced work ability than skilled employees. Unskilled work was significantly associated only with severely reduced work ability. Even though we found a significant correlation between unskilled occupations and physically strenuous work (r = 0.23), the relation between physical strain and work ability was not found. This is in contrast to other studies, which demonstrate that women's work environment continues to be physically demanding with aging, whereas men's becomes lighter [18,40]. On the other hand, working in unskilled occupations can involve other risk factors for work ability than physically strenuous work. Former research showed that high quality collaboration between unskilled staff and their leaders appears to be important [41]. Women in unskilled occupations lack alternative physically lighter and less demanding work [42]. Therefore, most probable outcome for individuals with severely reduced work ability in unskilled occupations may be sickness absence, followed by disability pensioning [43].

Much of the interest paid to sick leave and absenteeism has been based on the physical and psychosocial aspects of the work environment. Ongoing debates regarding sick leave and disability pensioning typically focus on the prevention of illness among healthy employees via workplace health promotion programs. Our results indicate that many employees report reduced work ability and this has a clear association with health and work type. Therefore, workplace health promotion efforts may be even more important for employees in poor health and in unskilled occupations to prevent further deterioration, allow their continued presence in the workforce, and thereby prevent permanent disability pensioning.

## Conclusion

Our results indicate that ageing, in addition to poor self-reported physical health and unskilled work, were the strongest factors associated with reduced work ability among female employees. Impact of work environment in general was visible only in univariate analysis.

## Authors' contributions

MG conceived and designed in consultation with the other authors the study, analyzed the data and drafted the manuscript, JFN contributed to the concept and design and statistical analysis of the data, IS collected the data, BL contributed to the concept and interpretation of the data, DB contributed to the concept, design, statistical analysis and interpretation of the data, and drafted the manuscript. All authors read and approved the final manuscript.
